# Integrating multi-omics insights into music and dance-based physical activity for cancer rehabilitation: implications for patient education and precision oncology

**DOI:** 10.3389/fspor.2025.1728168

**Published:** 2026-01-30

**Authors:** Yazhen Zhang, Yisheng Chen, Ke Wu, Zhaoyuan Huang

**Affiliations:** 1 School of Physical Education, Ningde Normal University, Ningde, Fujian, China; 2 Department of Otolaryngology, Ningde Clinical Medical College, Fujian Medical University, Ningde, Fujian, China; 3 Department of Otolaryngology, Ningde Municipal Hospital of Ningde Normal University, Ningde, Fujian, China; 4 Department of Otolaryngology, Pingnan General Hospital of Ningde Hospital Medical Group, Ningde, Fujian, China; 5 Kangwon National University, Chuncheon, Gangwon-do, Korea

**Keywords:** cancer rehabilitation, dance therapy, multi-omics, music therapy, neuroimmune modulation, physical activity, precision oncology

## Abstract

Music and Dance-Based Physical Activity (MDPA) is an emerging, exercise-centered therapy that integrates artistic expression with structured physical movement, offering significant benefits for cancer rehabilitation. By combining dance with music therapy, MDPA enhances motor coordination, emotional regulation, and physiological resilience. Advances in multi-omics technologies, such as genomics, proteomics, and metabolomics, have provided valuable insights into the molecular mechanisms underlying these benefits, establishing a solid scientific foundation for its clinical application. MDPA modulates neuroendocrine function, inflammatory signaling, and metabolic reprogramming, promoting immune balance and neuroplasticity. Omics-based analyses further reveal the regulation of genes related to stress response, cellular remodeling, and mitochondrial metabolism in patients engaged in music and dance programs. These findings suggest that MDPA is a promising strategy for precision rehabilitation, with the potential to complement conventional cancer treatments. Importantly, integrating MDPA into patient education could enhance its impact, as patients become more informed about the molecular mechanisms at play and how MDPA can complement traditional therapies. Future research should focus on establishing clear links between molecular changes and clinical outcomes, validating MDPA through multicenter trials, and creating personalized implementation frameworks to integrate it effectively into routine oncology care.

## 1 Introduction

Cancer remains one of the most formidable challenges to global health, with approximately 19.3 million new diagnoses and 9.9 million deaths reported in 2020 (1). This burden is expected to grow further as the global population continues to age and lifestyle-related risk factors accumulate (1). Although conventional therapeutic strategies such as surgery, chemotherapy, radiotherapy and the more recent development of immunotherapy have substantially improved patient outcomes, their limitations are equally evident (2–4). These interventions often result in profound physical toxicities including immunosuppression, fatigue and organ impairment, along with psychological consequences such as depression, anxiety and social role disruption. Such challenges highlight the urgent need for complementary strategies that address both physiological resilience and psychosocial well-being in cancer care (5).

The therapeutic use of music and dance has deep historical roots, with rhythm and movement employed in healing rituals since ancient times (6). Modern music and dance therapy, informed by psychology, kinesiology, and neuroscience, has gained traction in oncology since the 1990s, demonstrating the ability to alleviate cancer symptoms and improve quality of life. While high-income countries have integrated these therapies systematically, low- and middle-income regions still use them mainly in small-scale or exploratory programs.

Research shows that music and dance therapies benefit patients with various cancers, including breast, lung, and colorectal cancers (7). Music therapy reduces pain perception, modulates stress biomarkers, and lowers anxiety, while dance improves motor function, coordination, and body image (8). These therapies also promote social connectedness and enhance treatment adherence. However, issues like small sample sizes, heterogeneous study designs, and inconsistent protocols limit the strength of the evidence.

While short-term benefits are well-documented, the long-term biological and clinical effects are less understood. Personalized therapeutic frameworks are needed to address the diverse needs of cancer patients, influenced by disease stage, treatment regimens, and psychosocial factors (9). This review summarizes current research on music and dance therapies in oncology, emphasizing clinical outcomes, potential molecular mechanisms, and integration challenges. It also highlights the need for large-scale, cross-cultural studies to support global implementation and align with the broader vision of precision medicine and exercise-based strategies in chronic disease management (10).

## 2 Physiological and psychological mechanisms of music and dance therapy

**Figure 1:**
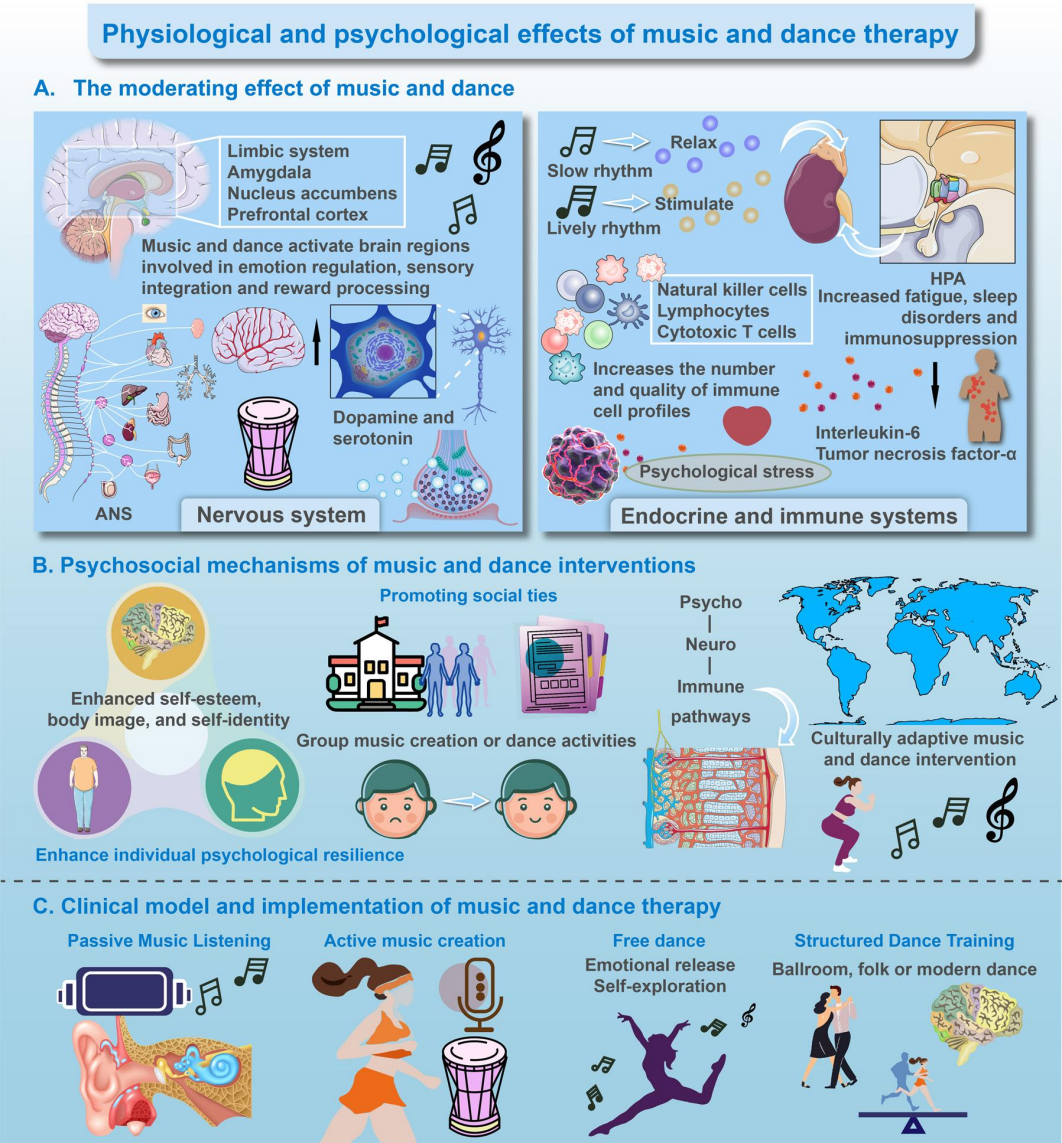
Physiological and psychological effects of music and dance therapy. **(A)** Physiological Foundations: Music and dance-based physical activity (MDPA) directly modulates core physiological systems. It activates key brain regions for emotion and reward processing, promotes neurochemical release, and regulates the autonomic nervous system and stress response (HPA axis). This foundational modulation leads to reduced inflammation, enhanced immune cell profiles, and overall physiological resilience. **(B)** Psychosocial Mediators: The physiological changes from **(A)** create a conducive basis for psychosocial benefits. Through group activities and individual expression, MDPA enhances self-esteem, body image, and social connectedness. These experiences, in turn, foster greater psychological resilience, forming a positive feedback loop that reinforces both mental and physical well-being. **(C)** Clinical Implementation: The foundational and mediating mechanisms are translated into practice through adaptable clinical modalities. These range from receptive to active interventions, which can be tailored to individual needs to ultimately promote emotional well-being, functional improvement, and enhanced quality of life.

Music and dance therapies stimulate areas of the brain associated with managing emotions, sensory processing, and reward, including the limbic system, amygdala, nucleus accumbens, and prefrontal cortex (Figure 1). The combination of rhythmic auditory stimulation and coordinated movement increases cerebral blood flow and metabolic activity in these regions, helping to alleviate symptoms such as anhedonia, emotional numbing, and motivational deficits in oncology patients. These therapies also promote neuroplasticity, aiding cognitive recovery and adaptive behavior (11). A core effect is the modulation of the autonomic nervous system, where slow-tempo music enhances parasympathetic activity, and dynamic dance sequences induce a controlled sympathetic activation. The resulting neurochemical changes, including increased dopamine and serotonin release, support improved mood, motor function, and sleep quality. Additionally, rhythmic entrainment and proprioceptive feedback optimize sensorimotor integration, enhancing both physical coordination and emotional regulation (12).

Music and dance therapies exert a systemic regulatory effect on the hypothalamic–pituitary–adrenal (HPA) axis, reducing cortisol levels and restoring adaptive diurnal rhythms, which mitigates chronic stress (13). This regulation alleviates fatigue while enhancing sleep quality and immune function in cancer patients (14, 15). Both interventions enhance immune profiles by increasing natural killer (NK) cells, lymphocytes, and cytotoxic T cells, which support tumor surveillance (Figure 1) (16). Furthermore, these therapies reduce pro-inflammatory cytokines like interleukin-6 and tumor necrosis factor-alpha, while promoting anti-inflammatory mediators, fostering a supportive immune environment. This dual modulation is especially beneficial for cancer patients undergoing immunotherapy, where immune balance is crucial.

Music and dance therapies offer nonverbal outlets for complex emotions, reducing psychological distress and fostering a sense of agency. Participants report improvements in self-esteem, body image, and identity, helping counter the existential impact of cancer. Group activities enhance trust, empathy, and social support, reducing isolation (Figure 1) (17, 18). These therapies stimulate reward and motivation systems, improving resilience, optimism, and proactive coping, which in turn enhances treatment adherence and long-term recovery. Tailoring interventions to cultural contexts increases engagement and benefits. For instance, group-based therapies may be more effective in collectivistic cultures, while individualized programs may suit more individualistic societies. Digital platforms offer an opportunity for cross-cultural application, delivering culture-specific content globally. Future research should investigate the effectiveness of culturally adapted protocols, particularly in comparative studies between China and other countries.

Music therapy can be passive (listening to music) or active (singing, playing instruments, improvisation), while dance therapy can be free-form or structured, enhancing coordination and balance. Both therapies can be adapted for individuals or groups to provide social support (Figure 1) (19). Optimal results are achieved with moderate-intensity sessions two to three times a week over eight to twelve weeks, with flexibility in program design to ensure safety and engagement. Integrating these therapies into interdisciplinary care models involving physiotherapists, psychologists, and oncologists is crucial for cancer rehabilitation. Digital and hybrid models increase accessibility, especially for patients with mobility challenges or in underserved areas.

## 3 Multi-omics insights into the biological impact of music and dance therapy

Transcriptomic analyses show that music and dance-based physical activity (MDPA) induces significant gene expression changes in cancer rehabilitation. RNA sequencing reveals alterations in genes related to neuroplasticity, cellular repair, and immune regulation, highlighting MDPA's ability to integrate multisensory, motor, and emotional stimuli for broad biological effects. These changes suggest MDPA provides both psychological and systemic benefits. MDPA upregulates genes linked to synaptic plasticity, including brain-derived neurotrophic factor (BDNF) and ARC, indicating improved cognitive resilience and rehabilitation adherence (20). MDPA also modulates immune pathways like NF-κB, JAK/STAT, and MAPK, downregulating pro-inflammatory cytokines and upregulating IL-10, potentially through vagus nerve-mediated cholinergic anti-inflammatory effects (21). Additionally, MDPA impacts stress-related genes, lowering cortisol and enhancing heart rate variability, suggesting HPA axis normalization and improved anti-tumor immunity. Preliminary findings indicate MDPA may also influence tumor cell death, angiogenesis, and cell cycle regulation, necessitating further clinical investigation.

Mass spectrometry-based proteomic analyses suggest that MDPA modulates proteins important for altered cell signaling, improved cytoskeletal dynamics, and enhanced oxidative stress responses, underpinning its multifaceted role in cancer rehabilitation (22). Specifically, MDPA affects several critical cancer-related pathways, including PI3K/AKT, mTOR, and ERK. The regulation of cell survival, metabolism, and immune activation by these pathways suggests a shift from pro-survival signaling to a state conducive to cellular homeostasis and immune surveillance (23). This model is functionally corroborated by two key findings. First, MDPA increases levels of immune-defense proteins such as complement components and immunoglobulins, which leads to enhanced opsonization and antibody-dependent cellular cytotoxicity, thereby strengthening innate and adaptive immune attacks on tumor cells. Second, MDPA upregulates the pro-apoptotic proteins. Together, these immunomodulatory and pro-apoptotic effects may synergistically improve systemic immune function and promote tumor cell death, particularly in the early stage of rehabilitation. Clinical correlation indicates an increase in lymphocyte count with a decrease in inflammatory markers, providing a systemic immunological validation of these proteomic alterations.

Concurrently, MDPA-induced changes in extracellular matrix proteins, such as fibronectin, collagen isoforms, and matrix metalloproteinases, point to a restructuring of the tumor microenvironment that likely impedes tumor invasiveness and angiogenesis (24). This “normalization” of the ECM may create a physical barrier against metastasis. Furthermore, the increased expression of mitochondrial and glycolytic enzymes indicates a systemic enhancement of energy efficiency. This bioenergetic boost is not exploited by the tumor, but is instead preferentially used to fuel the heightened activity of immune cells and to support tissue repair processes, thereby diverting metabolic resources away from tumor growth (25, 26).

Metabolomic profiling indicates that MDPA orchestrates a systemic metabolic shift, altering the metabolism of amino acids, lipids, and carbohydrates. Specifically, the improved turnover of branched-chain amino acids (BCAAs) directly supports protein synthesis and anaplerosis, thereby providing a molecular basis for the preservation of muscle mass, which is a critical aspect of cancer rehabilitation (27, 28). Simultaneously, the ameliorated lipid profile may reduce chronic inflammation and associated metabolic complications. Notably, elevated levels of neurotransmitter metabolites, including serotonin, dopamine, and *γ*-aminobutyric acid, suggest that MDPA modulates the gut-brain axis or neuro-immune circuits. This regulatory role could explain the observed improvements in mood and its positive impact on immune function. Epigenetic and miRNA analyses further substantiate the mechanism. The observed changes in DNA methylation and histone modification likely contribute to the reactivation of tumor suppressor genes and silencing of oncogenes, consistent with proteomic findings. Moreover, changes in microRNA profiles shed light on the specific molecular processes through which MDPA may exert its anti-cancer effects.

Microbiome sequencing indicates that MDPA enhances gut microbial diversity, specifically enriching beneficial genera such as *Bifidobacterium* and *Lactobacillus* (29, 30). These shifts correlate with elevated synthesis of short-chain fatty acids (SCFAs), which exert antitumor and anti-inflammatory effects while fortifying the gut barrier. Alterations in microbial composition also contribute to immune modulation by promoting regulatory T cell differentiation through enhanced antigen presentation and reduced cytokine production (31, 32). Taken together, these microbiome-mediated effects suggest that MDPA modulates the gut-immune-brain axis (15) and induces beneficial epigenetic reprogramming of host cells; these changes strengthen host defense, attenuate inflammation, and restore metabolic homeostasis.

## 4 Molecular mechanisms of music and dance therapy

**Figure 2:**
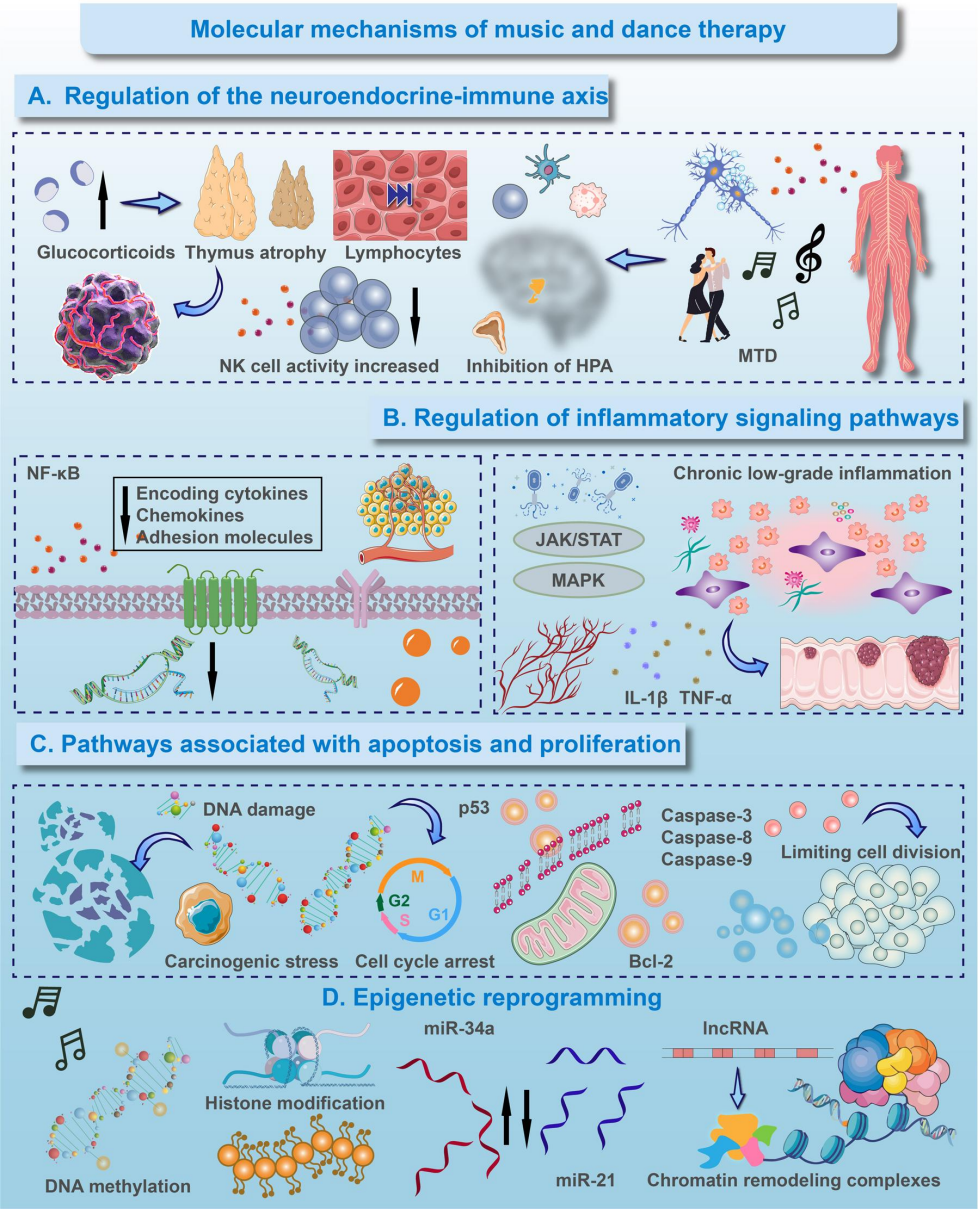
Molecular mechanisms of music and dance therapy. **(A)** Regulation of the neuroendocrine-immune axis. MDPA mitigates stress-induced immunosuppression by modulating the hypothalamic-pituitary-adrenal (HPA) axis, leading to reduced glucocorticoid levels. This, in turn, alleviates thymic atrophy, increases lymphocyte count, and enhances natural killer (NK) cell cytotoxicity. **(B)** Regulation of inflammatory signaling pathways. MDPA exerts anti-inflammatory effects primarily through the suppression of key pro-inflammatory pathways. It inhibits the nuclear factor-kappa B (NF-κB) signaling cascade, reducing the transcription of downstream cytokines, chemokines, and adhesion molecules. Concurrently, MDPA modulates the JAK/STAT and MAPK pathways, contributing to the attenuation of chronic low-grade inflammation driven by factors such as IL-1β and TNF-α. **(C)** Pathways associated with apoptosis and proliferation. In response to carcinogenic stress and DNA damage, MDPA promotes the stabilization and activation of the p53 tumor suppressor protein, leading to cell cycle arrest. Furthermore, it shifts the balance towards apoptosis by upregulating pro-apoptotic proteins and downregulating anti-apoptotic factors, thereby limiting uncontrolled cell division. **(D)** Epigenetic reprogramming. MDPA facilitates long-term adaptive changes through epigenetic modifications. These include alterations in DNA methylation patterns, post-translational histone modifications, and regulation of non-coding RNAs, collectively mediated by chromatin remodeling complexes to reinforce a favorable gene expression profile.

MDPA's regulation of the HPA axis and autonomic balance leads to specific molecular changes. Studies show that MDPA reduces cortisol levels, which is critical for preventing stress-induced suppression of immune responses like lymphocyte proliferation and NK cell activity (33). Rhythmic movement with sound enhances parasympathetic tone, activating the cholinergic anti-inflammatory pathway, which suppresses pro-inflammatory cytokines and supports immune function (34). This promotes NK cell cytotoxicity and enhances T cell responses, potentially improving cancer therapy outcomes. Thus, by co-modulating the neuroendocrine and immune systems, MDPA provides a valuable non-pharmacological adjunct to conventional cancer therapies (Figure 2) (35, 36).

MDPA may also modulate key inflammatory pathways like NF-κB, which is involved in tumor-supporting microenvironments (Figure 2) (37). By decreasing pro-inflammatory cytokines and restoring immune homeostasis, MDPA may improve treatment tolerance and efficacy, especially in combination with immunotherapy (38–40). However, the direct effects of MDPA on these pathways in human cancer patients remain speculative, based mainly on preclinical models. Additionally, MDPA may influence tumor cell fate through pathways like p53, involved in apoptosis and DNA damage responses (Figure 2) (41). Stress reduction via MDPA may enhance p53 activity, sensitizing cancer cells to chemotherapy and radiotherapy. MDPA's potential to modulate cell proliferation and induce apoptosis also suggests synergy with conventional cancer treatments.

Preliminary evidence indicates that MDPA could induce beneficial epigenetic changes, including DNA methylation and histone acetylation, potentially reversing tumor-promoting patterns (42). MDPA may influence microRNA expression, increasing tumor-suppressive miRNAs and reducing oncogenic miRNAs (43). These changes offer a non-invasive method to promote favorable gene expression in cancer survivors, supporting the integration of MDPA into precision oncology approaches. Future research combining multi-omics techniques will be crucial to validate these mechanisms and their clinical relevance. Incorporating MDPA into patient education can enhance understanding of these molecular processes, helping patients recognize the potential benefits of such therapies as complementary strategies to conventional cancer treatments.

## 5 Interdisciplinary integration and future clinical applications

### 5.1 Combining multi-omics data with clinical insights

Recent progress in multi-omics technologies (genomics, proteomics, and metabolomics) has deepened our understanding of MDPA's biological mechanisms in oncology rehabilitation (44). The combination of these technologies can create a more comprehensive view of how MDPA induces molecular changes that influence both physical and emotional aspects of cancer recovery (45). By merging omics data with clinical data, such as patient-reported outcomes and functional assessments, we can generate mechanistic insights that support evidence-based protocols for MDPA's clinical implementation (46).

The integration of wearable technologies enables real-time monitoring of physiological parameters such as heart rate variability, movement, and respiration (47). Combined with self-reported measures of mood, fatigue, and pain, these data provide a dynamic assessment of MDPA effects (48, 49). Integrating wearable and multi-omics data may yield models that elucidate MDPA mechanisms and identify predictive biomarkers for patient stratification. Artificial intelligence can further personalize therapy parameters to optimize efficacy and engagement (50, 51).

### 5.2 Enhancing conventional cancer therapies through MDPA

MDPA can enhance conventional cancer treatments by modulating neuroendocrine-immune systems, reducing inflammation, and improving immune responses (52, 53). For example, studies show that music therapy improved anxiety and mood in breast cancer patients undergoing chemotherapy (54). Notably, this study, like many in the field, involved a relatively small cohort and was not randomized, limiting the strength of causal inference. Immune response may be enhanced by rhythmic activity or activity that activates emotions, which in turn enhances immuno-oncology treatments and checkpoint inhibitors (55).

MDPA may also reduce cancer-related side effects like fatigue, cognitive dysfunction, and neuromuscular dysfunction, improving treatment tolerance and adherence (56). The neuroplastic effects of MDPA may help patients cope with treatment toxicity, such as neuropathic pain or nausea, while fostering better psychosocial outcomes (57). Integrating MDPA with other therapies, like psychotherapy or nutrition, could enhance rehabilitation, particularly in managing cancer-related fatigue and improving quality of life.

### 5.3 Precision strategies in MDPA implementation

Precision rehabilitation aligns MDPA interventions with a patient's specific molecular, physiological, and psychosocial characteristics (58). For example, MDPA can be customized for specific cancer types, treatment phases, and genomic markers, such as integrating oncological and nutritional expertise for personalized care during chemotherapy. Molecular subtyping can guide intervention selection, with personalized therapies addressing specific symptoms like inflammation or neuromuscular deficits. During active treatment, MDPA can focus on fatigue management, while in post-treatment phases, it can promote long-term health with aerobic or resistance training. Regular assessments and real-time data can ensure personalized care, enhancing treatment adherence and outcomes.

### 5.4 Overcoming barriers to clinical adoption

Widespread clinical adoption of MDPA requires standardized guidelines, multicenter trials, and long-term follow-up to establish its safety, efficacy, and cost-effectiveness (59). Demonstrating long-term cost savings through health economic evaluations will be critical for MDPA's integration into standard care protocols and securing insurance coverage (60). Public awareness, professional training, and interdisciplinary collaboration are key to overcoming barriers. Partnerships between healthcare providers, academic institutions, and arts organizations will expand MDPA's capacity and ensure high-quality care, driving innovation and improving patient outcomes.

## 6 Conclusion and future prospects

MDPA has gained strong empirical support in oncology rehabilitation, demonstrating benefits in alleviating cancer-related fatigue, reducing anxiety and depression, and improving pain management (61). It also enhances physical function and motor coordination, especially in patients recovering from chemotherapy, radiotherapy, or surgery (62). Emerging multi-omics research reveals that MDPA influences neuroendocrine activity, modulates immune-inflammatory responses, and promotes metabolic reprogramming, with measurable biochemical changes linked to psychological and physiological improvements. Its adaptability, alignment with the biopsychosocial model, and potential for patient-specific customization make MDPA a valuable non-invasive intervention. Additionally, group-based formats foster social engagement and emotional resilience, improving long-term quality of life (63).

However, current studies are limited by small sample sizes, varied demographics, and inconsistent methods, hindering the identification of clinically significant effects. Future research should focus on large-scale, multicenter trials with standardized protocols. Key areas of investigation include how MDPA can complement established oncological therapies, such as immunotherapy and targeted therapy, by enhancing immune responses or mitigating side effects like fatigue. Subgroup analyses, cost-effectiveness evaluations, and the integration of technologies like AI-driven motion analysis is crucial to advancing MDPA's role in personalized rehabilitation (64, 65). These efforts will deepen our understanding of MDPA's long-term impact on patient outcomes across diverse cancer treatments across diverse cancer treatments (66).
